# Drug Repurposing for Parkinson’s Disease: The International Linked Clinical Trials experience

**DOI:** 10.3389/fnins.2021.653377

**Published:** 2021-03-19

**Authors:** Simon R. W. Stott, Richard K. Wyse, Patrik Brundin

**Affiliations:** ^1^The Cure Parkinson’s Trust, London, United Kingdom; ^2^Parkinson’s Disease Center, Department of Neurodegenerative Science, Van Andel Institute, Grand Rapids, MI, United States

**Keywords:** Parkinson’s, neurodegeneration, disease modification, neuroprotection, drug repurposing

## Abstract

The international Linked Clinical Trials (iLCT) program for Parkinson’s to date represents one of the most comprehensive drug repurposing programs focused on one disease. Since initial planning in 2010, it has rapidly grown – giving rise to seven completed, and 15 ongoing, clinical trials of 16 agents each aimed at delivering disease modification in Parkinson’s disease (PD). In this review, we will provide an overview of the history, structure, process, and progress of the program. We will also present some examples of agents that have been selected and prioritized by the program and subsequently evaluated in clinical trials. Our goal with this review is to provide a template that can be considered across other therapeutic areas.

## Introduction

In April 1973, the U.S. Food and Drug Administration (FDA) approved the use of amantadine for alleviating the symptoms of Parkinson’s disease (PD). The story of how a single anecdotal patient-doctor interaction resulted in this antiviral therapy being approved for PD represents an excellent example of successful drug repurposing (Schwab et al., 1969; Hubsher et al., 2012). However, as with all of the currently available medications for PD, amantadine only provides symptomatic relief for individuals affected by the condition, temporarily alleviating the motor related features (bradykinesia, rigidity, and a resting tremor). Successful identification and widespread availability of disease-modifying agents that can slow or stop the slow progression of the disease represents a huge unmet worldwide need for the affected community of 6–10 million individuals, and for their families who are also greatly impacted by the condition. Drug repurposing currently shows promise for being able to identity therapeutics that will make a profound difference to the rate of progression of the disease.

Drug repurposing – sometimes referred to as drug *repositioning*, *reprofiling* or *re-tasking* – is a strategy of identifying novel uses for approved or investigational drugs that are beyond their original medical indication (Pushpakom et al., 2018). It is an efficient and appealing approach as it reduces number of required steps for clinical development, and thus lowers the amount of time and expense for taking a medicine through to regulatory approval. Given that the clinical profile and pharmacokinetics/dynamics of most approved drugs are already very well characterized, in many cases researchers can move directly into Phase II evaluations to explore signs of efficacy in the new indication of interest. With over 3,600 drugs available (US FDA, 2019), each of which is biologically active on one, or often more than one, biological targets, there is abundant opportunity for cross-indication testing. Numerous commercial and academic organizations around the world have now employed drug repurposing to accelerate drug development for their medical conditions of interest. Sophisticated drug evaluation processes have been established by key stakeholders; a successful and effective example of this is the international Linked Clinical Trials (iLCT) program which focuses on PD.

In 2010, The Cure Parkinson’s Trust (CPT) began planning the Linked Clinical Trials initiative, which later was renamed “the international Linked Clinical Trials” program. Initially the initiative was greatly influenced by the pioneering efforts of the 2003 “Committee to Identify Neuroprotective Agents for Parkinson’s” (CINAPS) program launched by Walter Koroshetz and colleagues (Ravina et al., 2003). CPT sought to assemble an international committee of experts of diverse, relevant skills at an annual 2-day meeting to examine agents suitable for repurposing that exhibit potentially disease-modifying properties in pre-clinical models of PD. The iLCT initiative involved the drafting of dossiers for each agent that was being considered for repurposing and, as detailed further below, contained information on the properties of the drug and the scientific and clinical basis of why it was potentially interesting to test in PD. Every year, over 20 different agents were presented to the members of iLCT committee (Table 1) who would then be tasked to prioritize them and thereby determine which of the evaluated therapeutics were most suited to move forward into clinical testing. Following this process, CPT was mandated to explore practicalities and mastermind the upcoming clinical trials of the prioritized treatments as selected by the iLCT committee. This approach has worked well and, to the present day, has largely maintained its structure, with regular committee expansion, since its initial formation. The iLCT program has now run for 9 years and has launched clinical trials exploring agents that target many different individual biological mechanisms that might modify the neurological decline in PD. In this review, we will briefly discuss the process of prioritization and present some examples of ongoing iLCT projects.

**TABLE 1:** The international Linked Clinical Trials Committee.

Chair: Prof. Patrik Brundin, Van Andel Institute, United States	
Prof. **Andrew Lees**, University College London, UK Prof. **Ted Dawson**, Johns Hopkins University, USA Prof. **Michael Schwarzschild**, Harvard University, USA Prof. **Caroline Tanner**, University of California, USA Prof. **Karl Kieburtz**, University of Rochester, USA Prof. **Roger Barker**, University of Cambridge, UK Prof. **Jeff Conn**, Vanderbilt University, USA Prof. **Howard Federoff**, University of California, USA Prof. **David Simon**, Harvard University, USA Prof. **Tim Greenamyre**, University of Pittsburgh, USA	Prof. **John Trojanowski**, University of Pennsylvania, USA Prof. **Tom Foltynie**, University College London, UK Prof. **Flint Beal**, Weill Cornell Medicine, USA Prof. **Mark Mattson**, NIH, USA Prof. **David Sulzer**, Columbia University, USA Prof. **Dimitri Krainc**, Northwestern University, USA Prof. **Mark Cookson**, NIH, USA Dr. **Brian Fiske**, The Michael J. Fox Foundation, USA Dr. **Camille Carroll**, Univ. of Plymouth, UK Prof. **David Devos**, University of Lille, France

## The iLCT Process

**FIGURE 1:**
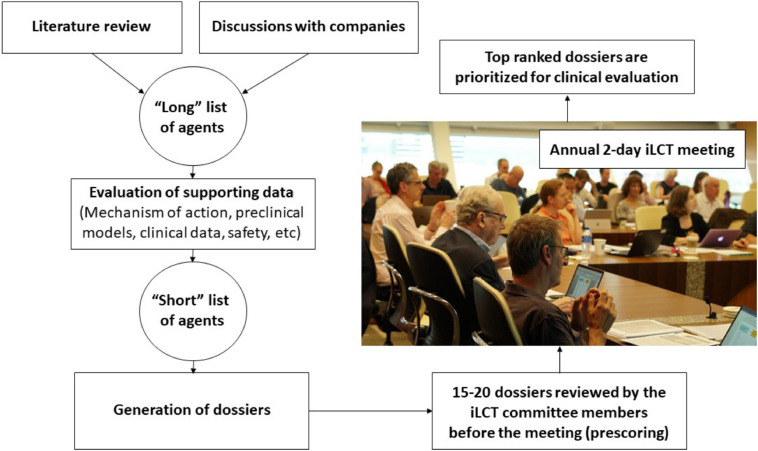
A flow chart of the process used in the iLCT scientific committee meetings.

The CPT research team and iLCT committee members continually review ongoing PD research and collect information on molecules that suggests that they might offer disease-modifying properties based on experiments in pre-clinical models of PD and sometimes also on information from epidemiological studies (Figure 1). Preceding the annual iLCT meeting in September, dossiers are written describing each drug, outlining potential strengths and weaknesses. Ideally, the agent has previously been tested in humans, with clear safety descriptions, pharmacokinetics, and pharmacodynamics data already available. There should also be evidence of acceptable bioavailability and CNS penetrance, unless the mode of action is proposed to involve a peripheral compartment such as the immune system or gut (although of course most manifestations of PD are related to changes in CNS pathology). In addition to promising results in models of PD (preferably in more than one model, and derived from multiple laboratories), any epidemiological data that may link exposure to the drug (when treating patients in the drug’s original therapeutic indication) with reduced incidence of PD, will be considered supporting evidence.

**FIGURE 2:**
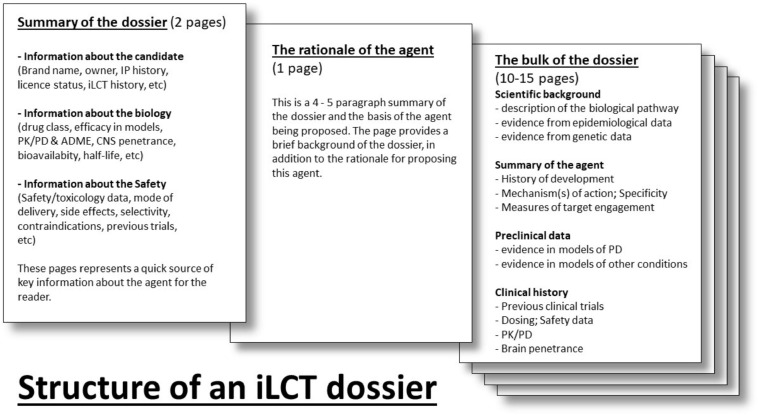
The layout of an iLCT dossier for each agent.

Each dossier is divided into separate sections (Figure 2). The first section provides a table summarizing necessary background information for the agent (including brand name, drug class, biological target, etc.). Next, there is an introductory section of 3–4 paragraphs that outlines the hypothesis and primary arguments justifying the clinical testing of this molecule in PD patients. In addition, a statement orientating committee members as to who was involved in generating the dossier, and what kind of clinical study, or PD patient subpopulation, might be considered. This section is followed by one that describes the main pre-clinical evidence and the clinical rationale that supports why the candidate drug should be considered. This starts with the “scientific background” section providing the reader with an overview of the biological pathway that is being discussed and how it relates to PD. The “summary of the intervention” delves into the nature of the agent (molecular weight, mechanism of action) and provides a history of its development. The “preclinical data” and “clinical development” sections outline experiments and clinical trials using the agent (or associated molecules where data are limited). The “summary” briefly explains why the agent deserves to be clinically tested in PD, and may include a note as to if the trial should focus on a specific type (e.g., genetic), symptom (e.g., cognitive dysfunction), or stage (early or late) of PD.

The dossiers are delivered electronically to the iLCT committee 2–3 weeks prior to the annual meeting, and they are tasked with providing a preliminary score for each dossier. These scores are used to triage the 20–25 dossiers down to the 15 that will be openly discussed amongst the committee members at the meeting itself. The iLCT meeting is held over 2 days at the Van Andel Institute in Grand Rapids (MI, United States) or at Cumberland Lodge, Windsor, United Kingdom. The Van Andel Institute partnered with CPT in 2012 to help coordinate the iLCT process. During each annual meeting, the final 15 dossiers that emerge from the triage process are presented individually by two designated committee members, then discussed by the whole committee, and then ranked alongside the other therapeutic candidates using a different scoring system (as compared with the triage stage). The average scores are calculated, and the top three-five drugs are considered prioritized to move forward into consideration for possible Phase II trials in PD patients.

The list of dossiers and the deliberations of the iLCT committee are not made publicly available. This is primarily for safety reasons, as some of the agents may have harmful effects if used incorrectly. In addition, some of the information discussed in the meeting can be sensitive (unpublished data) and not in the public domain. While the iLCT meeting is a closed-door meeting, an important aspect has always been a sense of inclusion. To this end, representatives of many of the major stakeholders are invited to attend the meeting (from major PD research charities to government organizations like NIH and NINDS). In addition, patient members of the PD research advocate community are also invited and actively engaged for their views on each dossier. All attendees of the meeting can provide feedback and comment on each dossier/agent being presented, but only the iLCT committee members provide scores.

Once a drug is prioritized, The Cure Parkinson’s Trust, Van Andel Institute, and partner organizations are then mandated to initiate clinical testing of that therapeutic. This process has led to seven completed trials and 15 molecules now being tested in 16 ongoing trials (one of which is a Phase III trial). In the following sections, we provide examples of drugs that have been prioritized by the iLCT committee. Specifically, we describe the history of the agent, the supporting evidence for its prioritization by the committee, and the current status of its clinical development for PD.

### GLP-1 Receptor Agonists

The first agent given top priority by the iLCT committee in 2012 was the long acting glucagon-like peptide-1 (GLP-1) receptor agonist exenatide (Bydureon; Brundin et al., 2013). This once-a-week, widely used injectable therapy for type 2 diabetes, stimulates glucose level–dependent release of insulin and β islet cell proliferation, whilst, at the same time, reduces β islet cell apoptosis (Buse et al., 2004). In addition to their insulinotropic actions, GLP-1 receptor agonists exhibit beneficial neuroprotective properties in both neurotoxic and synucleinopathy PD models (Harkavyi et al., 2008; Li et al., 2009; Liu et al., 2015; Chen et al., 2018; Yun et al., 2018; Zhang et al., 2019). GLP-1 receptors are present throughout the CNS, and exenatide can access the brain (Kastin and Akerstrom, 2003).

A randomized, single-blind Phase II study of exenatide in people with PD was initiated in 2010 at University College London, Institute of Neurology. Over 12 months of treatment, exenatide was found to be safe and well tolerated in people with moderate PD. In this unblinded study, the treatment also improved PD symptoms (based on the MDS-UPDRS) by an average of 2.7 points. This improvement was compared to a decline of 2.2 points in a parallel group of control patients who were on conventional PD treatment only (Aviles-Olmos et al., 2013). More remarkable; however, was a follow-up assessment of some of the participants in this first study which suggested that the observed beneficial effects persisted for at least 12 months after exenatide treatment had been terminated (Aviles-Olmos et al., 2015).

Following prioritization by the iLCT committee, a second Phase II study of exenatide in PD patients was initiated. This study was configured as a randomized, double-blind, placebo-controlled trial involving 60 individuals with PD. After 48 weeks of treatment, the exenatide-treated group exhibited a statistically significant reduction in the progression of their MDS-UPDRS III motor features compared to the placebo treated control group (Athauda et al., 2017). In doing so, the treatment met the predefined primary outcome of this trial. Additional *post hoc* analyses provided hypothesis-generating insights into clinical characteristics of the best responders to exenatide, and suggested that they tended to be younger at the time of disease onset and had a disease duration of less than 10 years at the time of the trial (Athauda et al., 2019b). A second *post hoc* analysis explored the use of neuronal-derived exosomes as a method of demonstrating target engagement and revealed changes in brain insulin, Akt, and mTOR signaling pathways consistent with increased stimulation of the GLP-1 receptor (Athauda et al., 2019a).

These results generated excitement within the PD community and a Phase III clinical trial has now been initiated (ClinicalTrials.gov, n.d. g). A total of 200 people with PD are now being recruited and randomized to be treated in a double-blind fashion with weekly administration either of exenatide or placebo, and being followed for 96 weeks, much longer than the previous studies mentioned above. The results are expected in 2024. In addition to this Phase III study, two other GLP-1 receptor agonists (that are each already approved for use in treating diabetes type II) are also in clinical trials in specific PD patient subgroups, and as a direct consequence of the iLCT program. A Phase II study of Liraglutide is currently being conducted in California (ClinicalTrials.gov, n.d. l), and involves 60 participants with PD and insulin resistance. A nationwide Phase II trial of Lixisenatide in 158 people with early stage PD is also being conducted in France (ClinicalTrials.gov, n.d. o). The results of these studies will be available in 2021/22.

One encouraging by-product of a large, robust drug repurposing program – such as the iLCT program – can be the stimulating effect that it can have on the wider research community. This phenomenon has demonstrated itself with the development of other GLP-1 receptor agonists that are currently being clinically developed for PD. For example, the biotech firm Neuraly is currently testing their novel pegylated long-acting GLP-1 receptor agonist, called NLY01 in a Phase II trial in PD (ClinicalTrials.gov, n.d. a). Other commercial companies are also evaluating their GLP-1 receptor agonists in Phase II trials for PD [these include the South Korean company Peptron’s PT320 (ClinicalTrials.gov, n.d. m)] and Novo Nordisk’s longer acting GLP-1 receptor agonist Semaglutide (ClinicalTrials.gov, n.d. h). One can conclude that as a result of the initial iLCT clinical trial involving exenatide, GLP-1 receptor agonists now represent a novel yet important class of potentially disease-modifying drugs for PD and, in the coming years, we will know more about their true potential and whether they are particularly suitable for long term disease-modifying use in certain subgroups of PD patients.

### Ambroxol

In 2009, the expectorant ambroxol hydrochloride (Ambroxol) was identified as a chaperone of the lysosomal enzyme β-glucocerebrosidase (GCase) in a screening study of FDA-approved drugs – (Maegawa et al., 2009). Ambroxol has been widely used in Europe as a treatment for respiratory diseases associated with mucus hypersecretion, and this new finding suggested that it could potentially be repurposed for PD. Genetic variations in the GBA1 gene – which encodes GCase – are some of the most common genetic risk factors associated with PD (Sidransky et al., 2009). Functionally, GCase is a lysosomal enzyme, but the mutated form of it becomes trapped in the endoplasmic reticulum, and is proteasomally degraded (Maor et al., 2013). This is the mechanism believed to result in lysosomal dysfunction in conditions (such as PD and Gaucher disease) that are associated with certain *GBA1* variants. In GBA1-associated PD, carriers present a similar phenotype to idiopathic PD, but they generally have an earlier onset of symptoms and there is an increased risk of cognitive impairment (Beavan and Schapira, 2013).

Following the discovery of its GCase chaperone function, Ambroxol was shown to raise CNS levels of GCase in transgenic PD mouse models, fibroblasts from patients with GBA-associated PD, and in the brains of non-human primates (McNeill et al., 2014; Migdalska-Richards et al., 2016, 2017). Ambroxol also improves the translocation of mutant GCase from the endoplasmic reticulum to the lysosome, increasing GCase activity in cells carrying GBA1 mutations (Maor et al., 2016). Furthermore, in mice overexpressing human α-synuclein, ambroxol treatment was found to decrease both α-synuclein and phosphorylated α-synuclein levels in the brain (Migdalska-Richards et al., 2016).

Based on these positive preclinical results, in 2014 the iLCT committee prioritized ambroxol for clinical evaluation in PD, and the “AiM-PD” (ClinicalTrials.gov, n.d. d) trial was initiated. This Phase IIa study involved 18 participants with PD given an increasing dose of ambroxol (up to 1260 mg per day) for 6 months. The results of the AiM-PD study were recently published (Mullin et al., 2020), demonstrating that ambroxol was safe and well tolerated in the PD cohort over 6 months of treatment, and that it significantly raised GCase protein levels in cerebrospinal fluid samples by approximately 35%, thereby providing evidence of target engagement. Clinical assessments indicated some benefit but, given the open label nature of the study and its shortness of duration (6 months), interpretations of those results must be handled with caution. A larger Phase III study is now being planned to assess the efficacy of ambroxol in PD.

Following the initiation of the iLCT study, additional clinical trials of ambroxol have been started. A Phase II study in London (Canada) is exploring ambroxol in 70 people with PD dementia (ClinicalTrials.gov, n.d. c; Silveira et al., 2019). In addition, the “Ambroxol in New and Early Dementia with Lewy Bodies (ANeED)” study is set to start in Norway this year (Rongve et al., 2020). This study will be a Phase IIa, multi-center study of ambroxol in prodromal and mild dementia with Lewy bodies. Ambroxol is also being evaluated over 12 months in 60 individuals with Gaucher disease in a Phase II trial (ClinicalTrials.gov, n.d. e).

### Ursodeoxycholic Acid

Ursodeoxycholic acid (or UDCA) is a secondary bile acid that is naturally synthesized in the liver and used medically – under the name ursodiol – for the treatment of gallstone disease and primary biliary cholangitis. A 2013 drug screening experiment – evaluating 2000 molecules for their rescue effect on mitochondrial dysfunction in Parkin (PARK2) patient-derived fibroblasts – highlighted UDCA and associated molecules for their ability to improve mitochondrial membrane potential and normalize ATP levels (Mortiboys et al., 2013). It was also reported to have a beneficial effect on the mitochondrial dysfunction associated with LRRK2 (G2019S) variants both in fibroblasts from variant PD carriers, and also in transgenic drosophila (Mortiboys et al., 2013, 2015). Additional models of PD have also revealed neuroprotective properties of UDCA (Amaral et al., 2009; Chun and Low, 2012; Abdelkader et al., 2016; Carling et al., 2020). A recent study suggested that unconjugated bile acids are increased in plasma from PD patients, tentatively suggesting that perturbations in bile acid metabolism are part of the underlying disease process (Yakhine-Diop et al., 2020).

In 2015, the iLCT committee gave UDCA top prioritization and a clinical trial was initiated by researchers at The University of Sheffield (Payne et al., 2020). The “UDCA in Parkinson’s study” (UP study) is a randomized double-blind, placebo-controlled study that began in early 2019. The trial involves 48 weeks of daily UDCA administration (30 mg/kg) in patients with early PD (less than 3 years since diagnosis). The results of this study are expected in 2021.

The UP study is not the only clinical trial exploring UDCA in PD – a small open-label, prospective, multiple-ascending-dose study of oral UDCA in five individuals with PD was recently completed (Sathe et al., 2020). The study found that the treatment was safe and well tolerated by PD patients, and that it was associated with modest increases in ATP and decreases in ATPase activity (based on MRS imaging which is also being explored in the UP study).

### Simvastatin

Statins are inhibitors of HMG-CoA reductase – the rate-limiting enzyme in cholesterol biosynthesis – but they have also exhibited neuroprotective properties, particularly in terms of the CNS-penetrant statin, simvastatin (Tong et al., 2018; Yan et al., 2018; Mattii et al., 2019; Simchovitz et al., 2019; Hanan et al., 2020; reviewed in Carroll and Wyse, 2017). It was also recently suggested that simvastatin treatment can reduce the risk of developing progressive supranuclear palsy and potentially delaying onset of symptoms (Bayram et al., 2020). Given the substantial supportive data available at the time, simvastatin was prioritized at the first iLCT committee meeting in 2012 and was put into clinical trial in a large nationwide Phase II study in the United Kingdom, called PD-STAT. This study involved recruiting 230 individuals with moderate PD, and daily administration of either simvastatin or placebo for 24 months (Carroll et al., 2019). The preliminary results of this trial were announced in September 2020 and indicated that simvastatin is safe in PD, but does not modify its course.

### Iron Chelation

In addition to the aggregation of α-synuclein, PD is also associated with the accumulation of iron in certain brain areas (Mochizuki et al., 2020). These elevated levels of iron may accelerate α-synuclein aggregation (Ostrerova-Golts et al., 2000), as well as directly cause oxidative stress and neurodegeneration (Deas et al., 2016). Given these associations, iron chelation has been considered as a therapeutic option for PD, with preclinical data providing a strong case for support (Finkelstein et al., 2016; Carboni et al., 2017; Das et al., 2017). The iron chelator deferiprone is another drug prioritized at the 2012 iLCT meeting. It is now in two large Phase II studies for PD (ClinicalTrials.gov, n.d. f, n.d. n). In addition, a second iron chelator was recently prioritized in the 2019 iLCT committee meeting. That compound is called ATH434, and it is being developed by Alterity Therapeutics (in collaboration with Takada Pharmaceuticals). Preclinical data for ATH434 suggest that the drug improves motor performance in multiple PD models (Finkelstein et al., 2017). Furthermore, Phase I safety, tolerability, and pharmacokinetics data in healthy human volunteers (Alterity Therapeutics, n.d.; ANZCTR, n.d.), support that the drug is safe and well tolerated. CPT and iLCT are now exploring ways to get this agent into a clinical trial in PD patients.

## Additional Considerations

The molecules discussed above represent a selection of the agents that have been prioritized by the iLCT committee and are now already in clinical evaluation for repurposing to PD. The process has not been without its challenges, however, and below we discuss some of the obstacles that can arise with drug repurposing.

### Right Target, Wrong Drug?

It might be easy to identify a biological pathway of interest and a drug to target it for a particular indication, but details like drug specificity, optimal dosing and CNS penetrance (in the case of PD) can impact drug repurposing efforts. An example of this has been inhibition of the non-receptor tyrosine kinase Abelson (c-Abl) for PD. cAbl is a ubiquitous kinase with a wide range of physiological functions (Hantschel and Superti-Furga, 2004). It becomes activated by DNA damage and cellular stress, and preclinical data indicated that not only was cAbl kinase activated in models of PD, but cAbl-inhibitors are effective in the laboratory at rescuing the neurodegeneration and associated motor issues (Hebron et al., 2013; Imam et al., 2013; Karuppagounder et al., 2014; Mahul-Mellier et al., 2014; Lee et al., 2018). Based on these preclinical findings, the iLCT committee prioritized the cAbl inhibitor nilotinib in 2013. However, before a trial was initiated, the results of a small, unblinded pilot study evaluating nilotinib in seven individuals with advanced PD dementia were presented at the 2016 Society for Neuroscience meeting. The results suggested potential benefits, and were later published (Pagan et al., 2016). These findings generated a great deal of excitement in the media, and also amongst patients. Given the enormous worldwide publicity of those initial open-label results, and the availability of the (albeit very expensive) drug which large numbers of PD patients had started to take off-label, large Phase II trials were set up to evaluate Nilotinib in PD: the PD-Nilotinib study (ClinicalTrials.gov, n.d. i) and Nilo-PD (ClinicalTrials.gov, n.d. j). The results of these studies have now been reported (Pagan et al., 2020; Simuni et al., 2020), and they suggest that not only did the agent have no effect on the progression of PD, but also that only a very limited amount of the drug actually reached the brain (CSF/serum ratio was 0.2–0.3%). cAbl may be an important target for PD, but it can be argued that nilotinib was probably not the best agent to evaluate this pathway in PD. Fortunately, cAbl inhibitors that access the brain more efficiently are now being clinically tested (for example, Sun/SPARC Pharma Advanced Research Company cAbl inhibitor K-0706 (ClinicalTrials.gov, n.d. k) and the Inhibikase Therapeutics cAbl inhibitor IkT-148009 (ClinicalTrials.gov, n.d. b).

### Dosing and Formulation

Another challenge of repurposing is the determination of dosing, which may differ significantly from that when used to treat the disease the drug was originally designed for. Preclinical models and non-human primate testing can aid in this process, but establishing potentially effective doses for humans can still impede rapid translation. Likewise, a new indication may require a novel formulation of (or method of administration for) the repurposed agent. We have experienced this with the ambroxol clinical trial program. The Phase II study discussed above required participants to self-administer 21 pills per day, on top of their current treatment regime of symptomatic medication. Heavy treatment requirements can affect compliance, especially in long-term studies. In such cases, reformulation may be required before further clinical progress can be effectively made.

### Intellectual Property

Expiration of patents can be a blessing and a curse for repurposing programs. In the absence of intellectual property (IP), initiating a repurposing clinical trial can become a more challenging undertaking. Manufacturing and, if necessary, encapsulation of clinical grade drug and placebo will add highly significant additional costs to a trial. However, if there is still life in a patent for a particular therapeutic, there is often a reason for interest from the holder of the IP. This can potentially result in financial support, or at least, access to drug and placebo to undertake a clinical trial for a new indication. The interest for the IP holder is in conducting a study, which may provide the proof-of-concept required for justifying investment in future derivatives of the agent. Such a path-finding study also provides the opportunity to evaluate and validate biomarkers for novel pathways that could be used to test the next generation molecules that are designed even more closely to match the requirements best suited to treat the disease.

### Repurposing ≠ Old Drugs

Drug repurposing should not be considered as limited just to clinically approved agents. New agents are continually entering the clinical trial process, providing a stream of novel therapies that could be “repurposed” for a disease area of interest. While IP holders may have very specific indications that they are focused on for economic reasons, drug repurposing programs like the iLCT can aid in catalyzing interest in these molecules in other indications. Biotech companies may have limited shareholder resources for conducting expensive clinical trials, but they often have enough clinical grade drug (and placebo) to consider additional medical areas if third parties (such as research charities) can organize funds for conducting a trial.

### Importance of the Patient Community Input

The primary task for the iLCT committee is appropriate drug selection. An important part of the deliberation is focused on safety and considerable thought is also given to patient wellbeing. At every iLCT meeting, patient advocates are invited as representatives for the PD community, and their input to the discussion is sought-after and greatly valued. For all drugs considered, special consideration must be given to whether any particular therapy is appropriate as a potential treatment for the cohort of interest – taking into account the practicalities of mobility and the predominant age bracket of the patients. Factors such as drug formulation and frequency of dosing are sometimes discussed. Unique insights can be gained from the lived experience provided by the patient advocates, and this can impact which of the agents considered by the committee are eventually selected to enter clinical trials.

## Conclusion

Drug repurposing has previously provided useful symptomatic therapies for the treatment of PD, and it is our hope that this approach will also provide a long-awaited means of delivering disease modifying treatments to the PD community. The iLCT initiative has been running for 9 years now, and while some of the drugs being tested may not necessarily be the ideal agent for each individual biological target being addressed, the drug repurposing approach does allow for a more rapid method of clinically evaluating the disease relevance of particular biological pathways/processes. As has been seen with the GLP-1 agonist examples, initiating such clinical trials can stimulate others to design and develop more appropriate molecules. It is our hope that these activities will result in novel disease-modifying therapies reaching the PD community sooner than the traditional approach to drug development.

## Author Contributions

All authors listed have made a substantial, direct and intellectual contribution to the work, and approved it for publication.

## Conflict of Interest

PB has received commercial support as a consultant from Axial Biotherapeutics, Calico Life Sciences, CuraSen, Fujifilm Cellular Dynamics, Inc., Idorsia Pharmaceuticals Ltd., IOS In review Press Partners, LifeSci Capital, LLC, Lundbeck A/S, and Living Cell Technologies Ltd. He has received commercial support for grants/research from Lundbeck A/S and Roche. He has ownership interests in AcouSort AB and Axial Biotherapeutics and is on the steering committee of the NILO-PD trial. SS and RW declare no financial, or any other, conflicts of interests and are employed by an international PD grant-giving charity.
